# Biological functions and research progress of eIF4E

**DOI:** 10.3389/fonc.2023.1076855

**Published:** 2023-08-03

**Authors:** Xiaocong Chen, Yang An, Mengsi Tan, Dongrui Xie, Ling Liu, Benjin Xu

**Affiliations:** ^1^ Department of Clinical Medicine, Fenyang College of Shanxi Medical University, Fenyang, China; ^2^ Department of Medical Laboratory Science, Fenyang College of Shanxi Medical University, Fenyang, China; ^3^ Key Laboratory of Lvliang for Clinical Molecular Diagnostics, Fenyang, China; ^4^ Department of Clinical Laboratory, Fenyang Hospital of Shanxi Province, Fenyang, China

**Keywords:** eIF4E, translation initiation, biological function, malignant tumor, antineoplastic drugs

## Abstract

The eukaryotic translation initiation factor eIF4E can specifically bind to the cap structure of an mRNA 5' end, mainly regulating translation initiation and preferentially enhancing the translation of carcinogenesis related mRNAs. The expression of eIF4E is closely related to a variety of malignant tumors. In tumor cells, eIF4E activity is abnormally increased, which stimulates cell growth, metastasis and translation of related proteins. The main factors affecting eIF4E activity include intranuclear regulation, phosphorylation of 4EBPs, and phosphorylation and sumoylation of eIF4E. In this review, we summarize the biological functions and the research progress of eIF4E, the main influencing factors of eIF4E activity, and the recent progress of drugs targeting eIF4E, in the hope of providing new insights for the treatment of multiple malignancies and development of targeted drugs.

## 1 Introduction

Translation initiation is the major regulatory stage of protein synthesis (1), which is an important rate-limiting link in protein translation (2). The first step in eukaryotic translation initiation that is dependent on the 5'-end cap structure of mRNAs is the assembly of the translation initiation complex eIF4F on the cap structure (3), and this complex recruits the ribosome to the 5'-end of the mRNAs (4). The eIF4F complex consists of the cap binding protein eIF4E, the large scaffolding protein eIF4G and the DEAD-box protein eIF4A. EIF4E can bind to the cap structure (5), recruiting eIF4G and eIF4A (2, 3, 6, 7).

*EIF4E* and *eIF4G* are oncogenes whose overexpression promotes cell transformation. Studies reported that *eIF4E, eIF4G* and *eIF4A* genes showed increased amplification or transcription in various human cancers (4). The overexpression of eIF4E precisely elevates the translation of mRNAs associated with tumor growth and invasion (8), making it an important target for tumor therapy. Xu et al. showed that reducing the levels of these translation factors, which have long been considered housekeeping genes, does not interfere with normal development or cell physiology, but is significantly associated with carcinogenesis, supporting the idea that eIF4E activity is critically involved in cancer development (4). Recent studies have shown that eIF4E can inhibit obesity and fatty liver caused by high-fat food. Therefore, the relationship among obesity, canceration and eIF4E may become a new direction for the study of eIF4E.

The action mechanism of eIF4E in promoting malignant transformation of cells has triggered wide attention, and some progress has been made so far. In this paper, the biological functions, activity influencing factors, and the research status of eIF4E in the treatment of tumors are reviewed.

## 2 Structures of eIF4E

**Figure 1:**
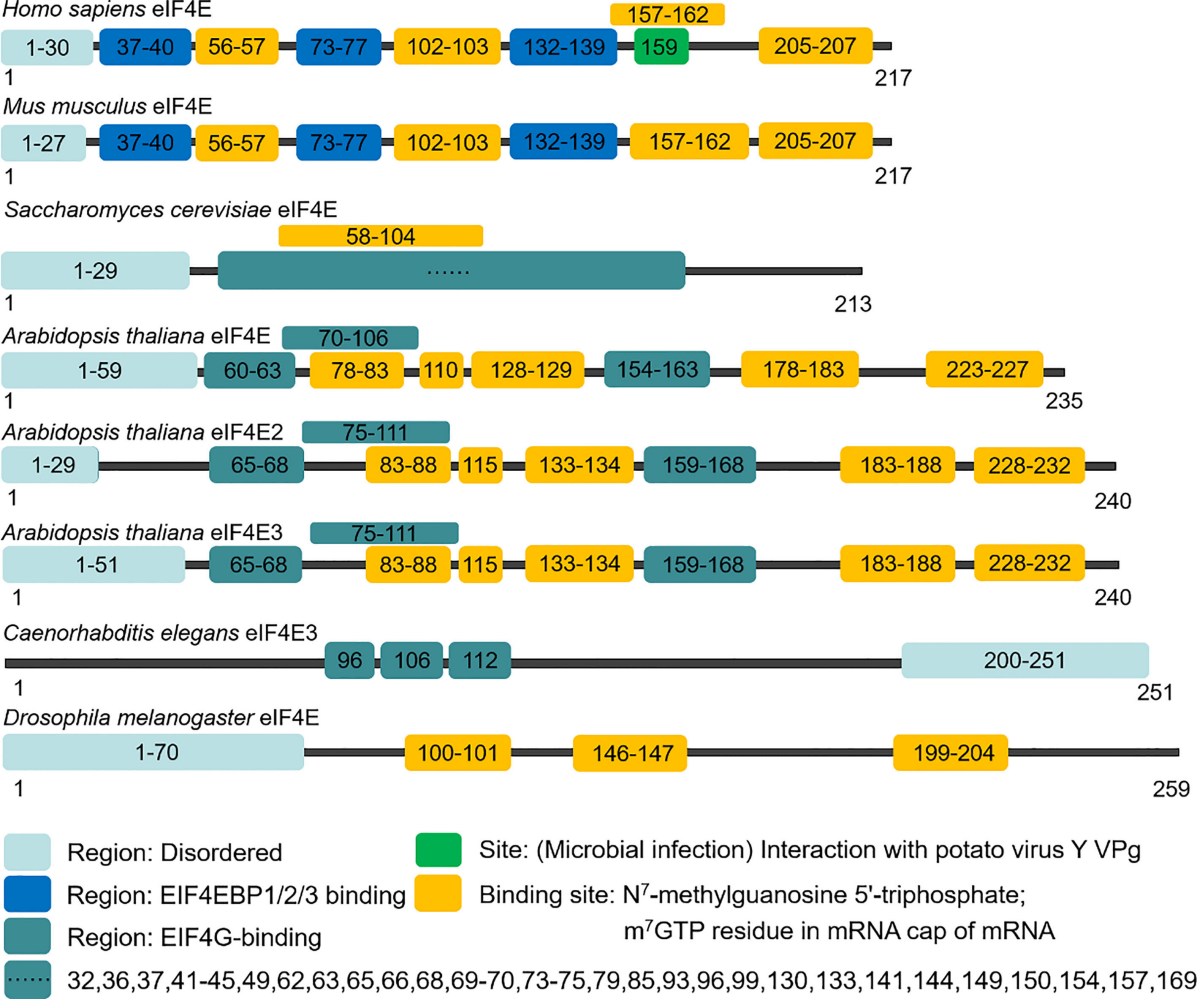
Domain organization of eIF4E.

**Figure 2:**
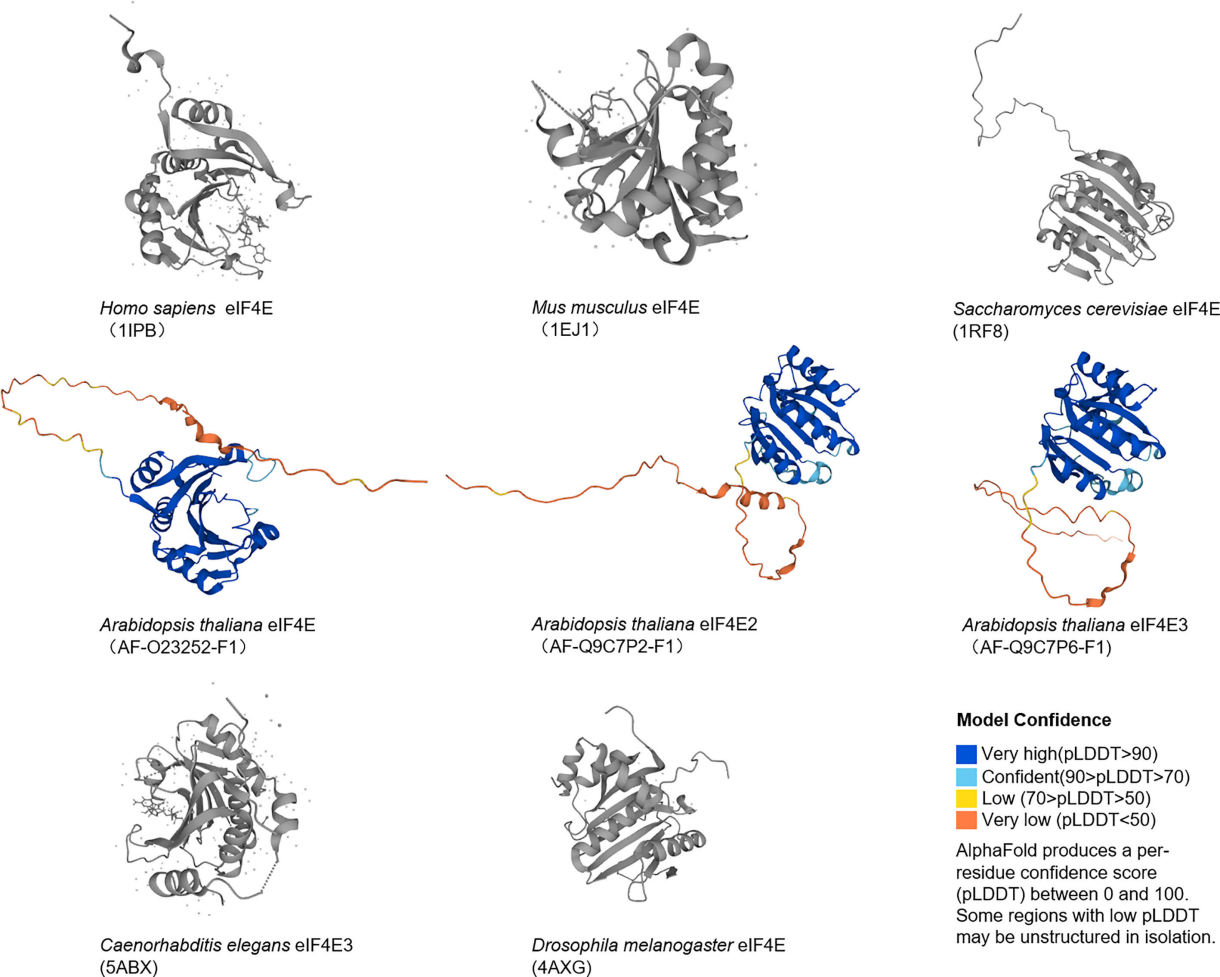
Structures of eIF4E.

The *eIF4E* gene, located on chromosome 4q21.25, encodes a conserved protein with a molecular weight of 25 kDa that can bind to the 5' end cap structure of mRNAs. In mammals, there are three members in the eIF4E family, eIF4E1, eIF4E2 and eIF4E3, which differ in structures, functions and expression patterns. EIF4E1 (eIF4E) is present in all eukaryotes (**Figures 1**, **2**), and orthologs of eIF4E2 appears to exist only in metazoans, while those of eIF4E3 are found only in chordates. EIF4E2 is ubiquitously expressed, with the highest levels in testis, whereas eIF4E3 is expressed only in skeletal muscle, heart, spleen and lung. Compared with eIF4E1, eIF4E2 and eIF4E3 respectively have some functions of eIF4E1. EIF4E2 cannot combine with eIF4G to initiate translation, and eIF4E3 cannot combine with 4EBPs (9). Three-dimensional structural analysis showed that there are two main binding sites on the surface of eIF4E: cap binding site and eIF4G (or 4EBPs) binding site. The concave face of eIF4E contains a concave hydrophobic pocket assembled by eight β-sheets and three α-helices. The cap binding site is on the basal face of the pocket, and the cap structure is stacked between two highly conserved tryptophan residues. EIF4G or 4EBPs specifically bind to the backside bulge of eIF4E, both of which share and competitively bind to this region (10, 11).

## 3 Biological functions and associated interacting proteins of eIF4E

**Figure 3:**
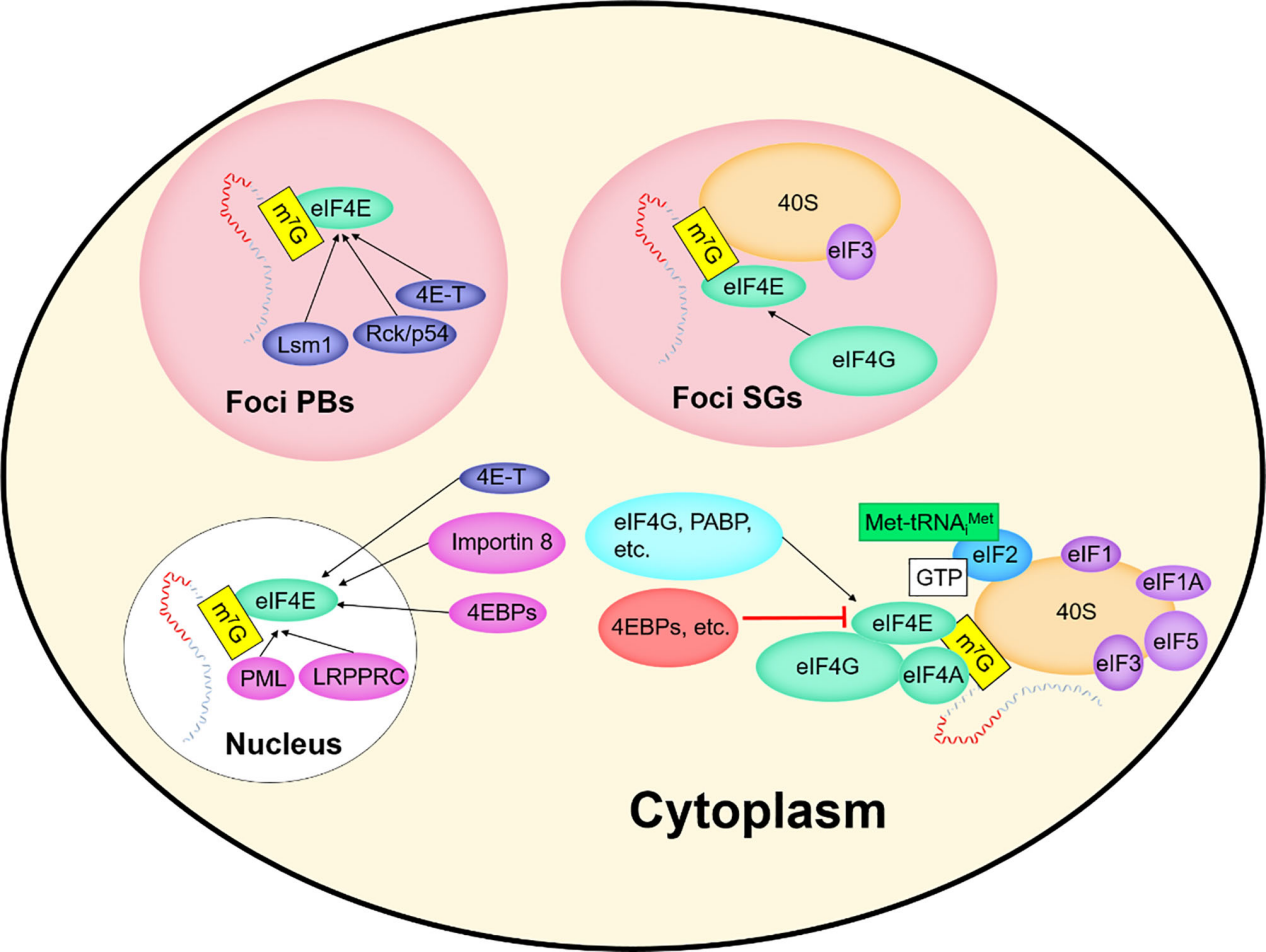
EIF4E and interacting proteins.

EIF4E plays a key role in mRNAs translation. In this paper, we associate eIF4E with the interacting protein 4E-IPs and focus on some functions of eIF4E: improving the translation efficiency of some mRNAs in the cytoplasm, mediating the nuclear export of mRNAs containing specific elements, and playing a role in the cytoplasmic foci. The activity and biological specificity of eIF4E depend on its interaction with different 4E-IPs. Over the years, about 47 different 4E-IPs have been found (12), which play an auxiliary role in the different functions of eIF4E (**Figure 3**).

### 3.1 EIF4E and nucleocytoplasmic transport

EIF4E is expressed in both nucleus and cytoplasm, with up to 68% of eIF4E distributed in the nucleus (13). In the nucleus, eIF4E binds the m^7^GpppN cap structure of transcripts and is solely responsible for regulating the nuclear export of eIF4E--dependent mRNAs, a class of mRNAs which mainly encode proteins related with proliferation and survival, such as Cyclin D1 (13–16) (**Table 1**), thus increasing their cytoplasmic concentration and protein production (14, 16, 25). The 3'UTR of this class of mRNAs have a structure of approximately 50 nucleotide units in length, which is termed the eIF4E sensitive element (4ESE) (14, 15). These eIF4E-dependent mRNAs are termed weak mRNAs after they are transported to the cytoplasm. Overexpression of eIF4E can alter the composition of the nuclear pore complex (NPC), increasing nuclear export of eIF4E-dependent mRNAs (26).

**Table 1:** Proteins translated by weak mRNAs.

Name	Function
Cyclin D1	It makes cells in G1 phase enter S phase by allosteric regulation (17).
c-Myc	It promotes cell cycle progression, transforming cells in G1 phase into S phase, and regulates cell apoptosis (18).
FGF2	Exogenous FGF2 is activated by binding to tyrosine kinase receptors of FGFRs, which leads to cell proliferation or migration, and endogenous FGF2 can be anti-apoptotic by binding to apoptosis inhibitor 5 (API5) (19).
VEGF	It stimulates endothelial cell proliferation and new blood vessel formation (20).
ODS	As a rate-limiting enzyme in polyamine synthesis, it regulates cell proliferation, apoptosis and senescence (21).
Bcl-2	Associated with chromosomal translocations, it can inhibit cell death (22).
Survivin	It has no enzymatic activity and helps to align the chromosomes correctly during mitotic metaphase. As one of the human inhibitors of apoptosis (IAP), it interacts with XIAP to inhibit apoptosis, and can also regulate mitochondrial dynamics and affect cell metabolism (23).
bFGF	It preserves the viability of epithelial cells, endothelial cells, smooth muscle cells and nerve cells, and inhibits their apoptosis (24).

The transport mechanism of eIF4E-dependent mRNAs is different from that of general mRNAs. mRNPs exported by eIF4E differ from general mRNPs in the export pathway, as eIF4E and eIF4E sensitive mRNAs(eIF4E-dependent mRNAs) are not associated with general export factors such as TAP/NXF1 or REF/Aly (27, 28). Cofactors involved in eIF4E export in mammalian nuclei include LRPPRC proteins and the export receptor CRM1 (25). mRNPs exported by eIF4E consist of eIF4E, mRNA containing the 4ESE element, LRPPRC, and other components, but not REF/Aly (26).

CRM1 mediates the nuclear export of hundreds of different functional proteins, including many tumor suppressors, cell cycle regulators, and many mRNAs. Inhibition of CRM1 with Leptomycin B may abrogate the export of eIF4E-dependent mRNAs, suggesting that eIF4E may interact with CRM1 and eIF4E-dependent mRNAs transport is mediated by CRM1 (25, 29). GST pull-down assay proved that CRM1 could directly bind LRPPRC and act as an export receptor to form LRPPRC-eIF4E-mRNA-CRM1 complex (25). LRPPRC could immunoprecipitate eIF4E, and knockdown of LRPPRC reduced the ability of eIF4E to immunoprecipitate with 4ESE RNA in the nucleus, which directly suggested that eIF4E could interact with LRPPRC (25, 27).

### 3.2 EIF4E and translation

#### 3.2.1 The mechanism of eIF4E in translation

**Figure 4:**
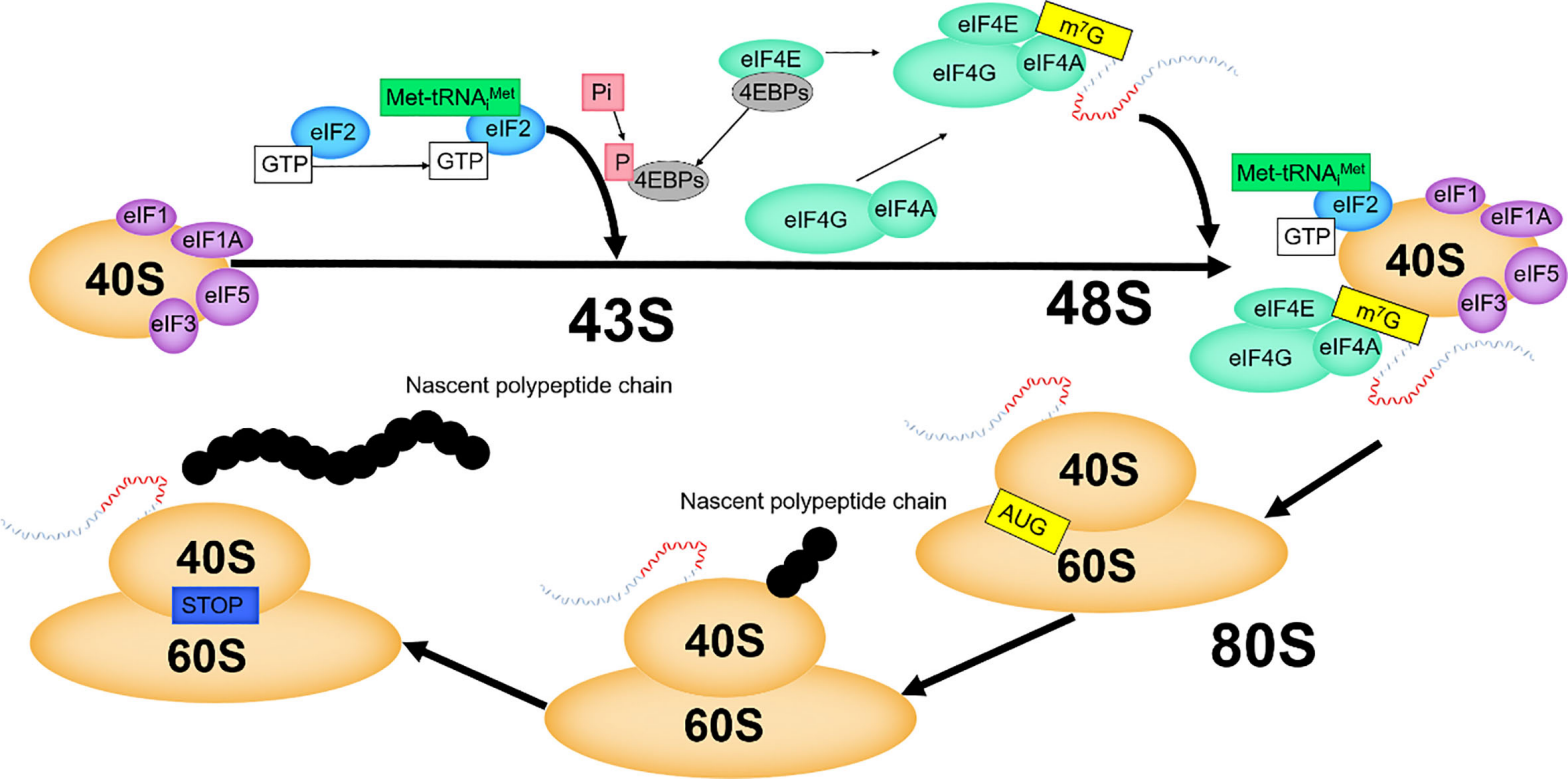
The mechanism of eIF4E in translation initiation.

In eukaryotes, translation is initiated by decoding AUG in mRNAs, which is executed by the 48S pre-initiation complex (PIC) composed of the mRNAs, eIF2/GTP/Met-tRNA_i_ ternary complex (TC), the small subunit of the ribosome, and the eIF4F complex. Then, 48S PIC combines with the large ribosomal subunit and assembles into the initiation complex. Subsequently, translation enters the elongation cycle (**Figure 4**).

EIF1, eIF1A, eIF3 and eIF5 promote the recruitment of TC to the small ribosomal subunit, and these factors then assemble to form 43S PIC (30, 31). In eukaryotes, eIF4F binds cap structure to activate mRNAs, which is crucial (30, 31). EIF4F promotes mRNAs to combine with the preassembled 43S PIC, forming 48S PIC (30, 31). By binding with the cap structure, eIF4E brings eIF4A to the 5' end of mRNAs, and makes eIF4A exert helicase activity to open the secondary structure of the 5' end of mRNAs. The interaction of eIF4G with eIF1, eIF3 and eIF5 plays an important role in facilitating the recognition of the 5' end of mRNAs by 43S PIC. The helicase activity of eIF4A is increased by the interaction of eIF4G with eIF4A (32). The eIF4E-eIF4G interaction is a key step in mRNAs recruitment, and this node is regulated by 4EBPs (33). After scanning from the 5' end to the 3' end of the mRNAs and positioning the start codon AUG (6), the 48S PIC changes from an “open” to a “closed” conformation to prevent scanning and binds to the 60S large subunit, which, with the release of initiation factors, eventually generates the 80S initiation complex with translation elongation capacity (34).

#### 3.2.2 EIF4E improves translation efficiency

Due to the limited expression of eIF4E and its binding to inhibitory 4EBPs, the active of eIF4F complexes is normally limited. Therefore, intracellular mRNAs must compete for binding to eIF4F to initiate translation (35).

The “availability” of mRNAs in the cytoplasm varies considerably, with the length and structure of its 5'UTR significantly affecting translation efficiency (36). According to the “availability”, mRNAs can be divided into strong mRNA and weak mRNA. The 5'UTR of weak mRNAs are long and complex, with stable secondary structures, which hinders the association between the 43S PIC and mRNAs, and also the scanning for the 5'UTR. Weak mRNAs require RNA helicases (such as eIF4A) to open the secondary structure, so the translation efficiency is low (37). Proteins such as VEGF, FGF2, c-Myc, ODC, Cyclin D1, Bcl2 and Survivin are translated from weak mRNAs (35, 36, 38), and most of them can promote the development of cancer. Weak mRNAs are more susceptible to regulation by eIF4E than other mRNAs and are known as eIF4E sensitive genes (13), and they are less translationally competent when eIF4F complex formation is restricted (36). The 5'UTR of strong mRNAs are short and simple, with low GC contents and less stable secondary structures, which can be effectively scanned to initiate start codon recognition. For example, β-actin and GAPDH encoded by strong mRNAs (35, 36), are efficiently translated even when eIF4F is limiting.

EIF4E in the cytoplasm is a translation enhancer of weak mRNAs, especially during cellular stress and proliferation (15, 16). When the levels of eIF4E are limited, the effect on the translation of strong mRNAs is minimal (36). When the levels of eIF4E are increased, the translation of weak mRNAs is disproportionately increased, there is a nonlinear relationship between the increase of eIF4E and the increase rate of weak mRNAs translation. With the increase of eIF4E, the increase rate of weak mRNAs translation is variable rather than constant (35, 36). Numerous studies have shown that the elevation of eIF4E levels preferentially enhances the translation of weak mRNAs (35), leading to tumorigenesis and metastasis.

### 3.3 EIF4E and cytoplasmic foci

The processing, transportation, degradation and translation of mRNAs are essential for gene expression regulation (39, 40), and these processes are controlled by specific RNA-binding proteins (RBPs) (39, 40). Many RBPs bind to mRNAs and assemble into mRNPs (41). After being exported from the nucleus, some mRNPs are transported to specific regions of subcellular localization, cytoplasmic foci (40, 42). Non-membrane-enclosed cytoplasmic foci, such as Stress Granules (SGs) and processing bodies (P-Bodies, PBs), dynamically sequester untranslatable mRNPs into compartments distinct from the surrounding cytoplasm. Both cytoplasmic foci are associated with translation events that affect cell survival (40). Representatives of the various enzymes required for the breakdown of mRNAs into their constituent parts including a deadenylase, a decapping enzyme, and an exonuclease, are concentrated in the cytoplasmic foci (43).

**Figure 5:**
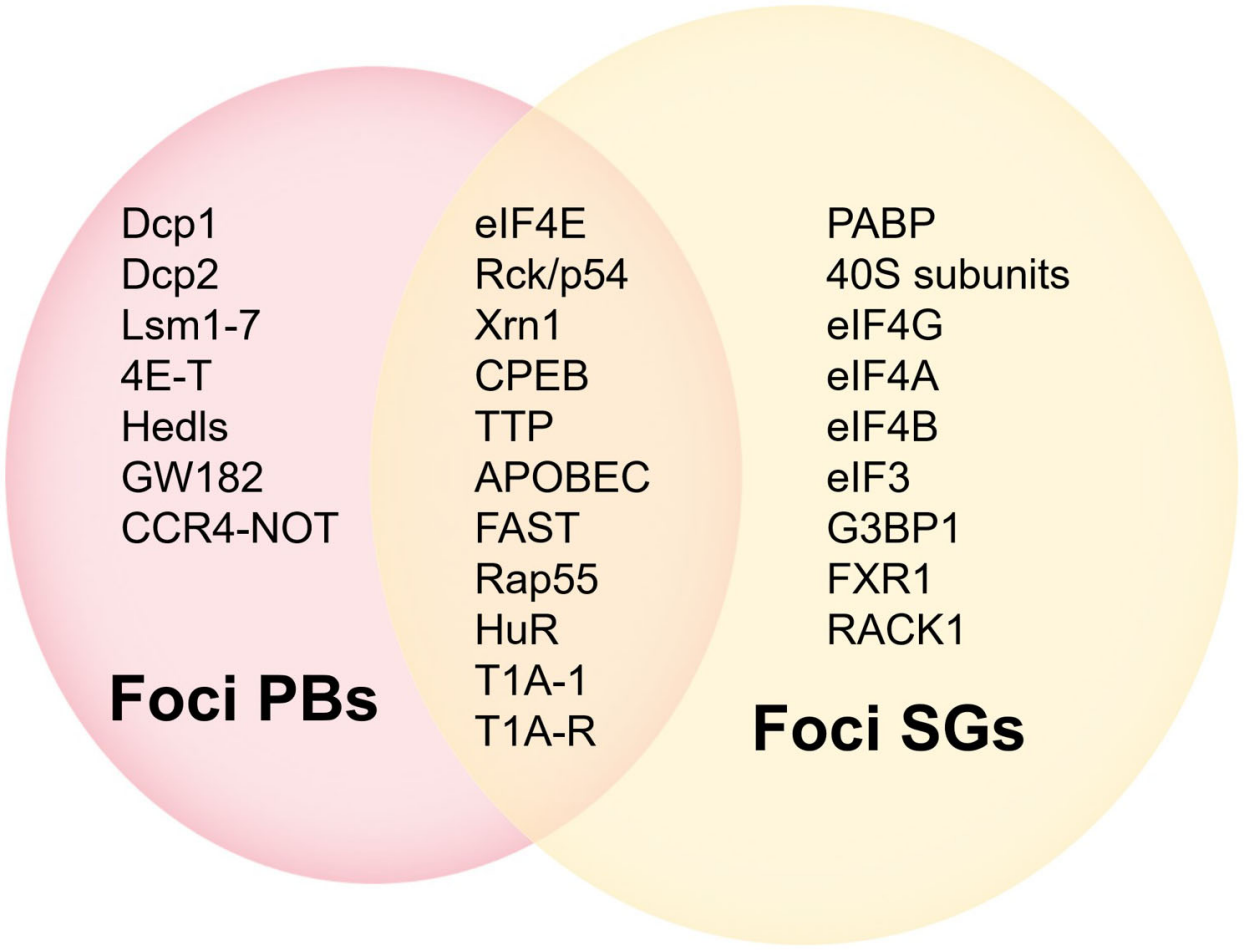
Comparison of components in PBs and SGs.

PBs differ from SGs in composition and function (**Figures 3**, **5**) (44, 45), but share some components and attributes, including eIF4E, mRNAs, and RBPs. Several components contained in PBs, such as eIF4E-T, Lsm1 and Rck/p54, are required for eIF4E accumulation in PBs. However, PBs lack SGs-associated eIF3, PABP, small ribosomal subunits, and many signaling proteins, and eIF4G accumulates only in SGs (40, 46, 47). Tryptophan residues needed for recognizing mRNA cap structure are not essential for the recruitment of eIF4E to PBs or SGs, and those required for protein-protein interactions are critical for eIF4E accumulation in cytoplasmic foci (46).

PBs are RNPs aggregates that contain a variety of proteins associated with mRNAs degradation (46, 48). PBs are associated with miRNA induced translation silence and siRNA induced mRNA degradation (43, 46). PBs can also act as a “storage depot” for mRNAs, where the stored mRNAs can be recycled under certain conditions (48). Among the new studies, researchers have identified two decapping activators, Dhh1p and Pat1p. Approximately only 10% of cells with a deletion of both proteins can form PBs, rendering the cells unable to normally inhibit the translation of some of the proteins that should not be expressed. The human homologue of Dhh1p is Rck/p54, which functions to inhibit translation *in vitro* (49). However, eIF4E can contact with 4E-T and Rck/p54 molecules in PBs *in vivo*, and this implies that the role played by eIF4E in cytoplasmic foci is also relevant to translation. PBs are rich in mRNAs degrading enzymes and contain eIF4E but not eIF4G, suggesting that eIF4E may play an early role in the transition from active translation to degradation of mRNAs. Moreover, due to the protection of the cap structure by eIF4E, some mRNAs, although their translation were repressed, may not be immediately targeted for degradation in the 5' to 3' direction (47).

Under certain non-physiological conditions in higher eukaryotes, such as heat or chemical stress, inactive mRNPs accumulate in the cytoplasm and SGs appear. SGs contain most of the components of 48S PIC but lack eIF2, which are called “abnormal aggregates of 48S PIC” (46, 47). SGs affect translation and stability of mRNAs and are associated with apoptotic. Disrupting eIF4F complexes, such as interfering with eIF4E activity, can lead to SGs formation.

The above studies suggest a novel role of eIF4E in mRNAs translation in cytoplasmic foci.

### 3.4 EIF4E interacting proteins

#### 3.4.1 LRPPRC and importin 8

LRPPRC is a leucine rich pentapeptide repeat protein implicated in the nuclear export of eIF4E. LRPPRC acts as a specific factor to recruit mRNAs containing 4ESE in the nucleus, and can directly bind eIF4E and 4ESE-containing mRNA through the N and C termini respectively, and binds CRM1 at the same time, thereby transporting the complex to the cytoplasm through the nuclear pore. LRPPRC is a central component of this mRNAs export pathway and is the first factor to function in eIF4E-sensitive mRNAs selection (25, 27). LRPPRC mediated nuclear output requires the integrity of its binding site with eIF4E (27).

EIF4E nuclear import is directly mediated by importin 8 and is competitively regulated by the m^7^G cap structure and importin 8 (50).

#### 3.4.2 4EBPs and 4E-T

4EBPs, widely distributed in the nucleus and cytoplasm, are eIF4E binding proteins and usually inhibit the role of eIF4E in translation. 4EBPs share and competitively bind to a common eIF4E binding site with eIF4G (10, 11), preventing their assembly into eIF4F. 4EBP1 and 4EBP2 are expressed in most tissues, while 4EBP3 shows a more limited expression pattern (51). Binding of 4EBPs to eIF4E is regulated by their phosphorylation status. Non-phosphorylated 4EBPs (active) bind to eIF4E with high affinity and phosphorylated 4EBPs (inactive) can not bind to eIF4E (52–54), and the expression of phosphorylated 4EBPs is associated with malignant progression and poor prognosis. Deletion mutations of 4EBPs genes are frequently found in patients with pancreatic cancer and head and neck cancer, suggesting a tumor-suppressive role of 4EBPs in some cancers (4).

4E transporter (4E-T), an eIF4E binding protein (4EBPs) present in PBs, represses translation and promotes mRNAs degradation and interacts with factors including DDX6, Lsm14 and the Lsm1-7-PAT1 complex (55, 56).

#### 3.4.3 PRH

Proline-rich homeodomain proteins (PRH) are composed of a proline-rich N-terminal domain, a central homeodomain that binds to specific DNA sequences, and an acidic C-terminal domain. This protein is encoded by a haematopoietically expressed homeobox (*HHEX*) gene (57), and acts on eIF4E in the nucleus. PRH is a regulator of transcription and translation, and plays an important role in the control of cell proliferation and differentiation (58), early embryonic patterning and hematopoietic processes (59), and is essential for forebrain, liver, and thyroid development. PRH is highly expressed in pluripotent erythromyeloid and B cell progenitors and is downregulated during differentiation of most hematopoietic lineages (60). In addition to hematopoietic cells, PRH is expressed in a limited set of tissues in the adult, including myeloid cells, lung, thyroid, and liver tissues, and has been localized as a tissue-specific regulator of eIF4E. Proline-rich N-terminal domain is essential for the inhibition of myeloid cell proliferation and cell transformation (58). PRH can directly interact with eIF4E through a conserved binding site and inhibit the transformation and growth promotion function of eIF4E by inhibiting its mRNA transport activity (61).

#### 3.4.4 PML

The promyelocytic leukemiaprotein, PML, acts on eIF4E in the nucleus. The integrity of PML nuclear body depends to some extent on the integrity of eIF4E nuclear body, whereas the integrity of eIF4E nuclear body is not related to PML (62, 63). PML contains three cysteine-rich zinc-binding domains, called RING and B-box (B1 and B2), which are required for the transformation suppressive and pro-apoptotic functions of PML (64). PML binds to the back of eIF4E, including Trp^73^, using a region around the first zinc binding site of the RING domain. The RING is essential for some physiological functions of PML, including nuclear bodies formation, growth inhibition and apoptosis (62, 65). Mechanism of PML binding to eIF4E as detailed later in “intranuclear regulation of eIF4E”.

#### 3.4.5 EIF4G and eIF4A

EIF4G is a large scaffold protein associated with DEAD-box proteins, with domains that bind to mRNA, eIF4E, eIF4A, poly (A)-binding protein (PABP), eIF3, eIF1, and eIF5 (2, 3, 5, 6).

EIF4A is a protein with a molecular weight of 44kDa and an ATP-dependent helicase belonging to the DEAD-box protein family, which is thought to unwind the secondary structure of mRNAs (4), facilitate scanning and exposure of the start codon of mRNAs (30, 36, 66).

## 4 Regulation of *eIF4E* expression

EIF4E is often highly expressed in tumor cells, and few studies have investigated how the levels and activity of eIF4E are elevated in malignant cells.

### 4.1 Increased gene copy number

Sorrells and Haydon et al. investigated *eIF4E* gene copy number by using western blot and PCR. Overexpression of eIF4E in breast cancer samples was associated with increased gene copy number, which was not detected in benign breast tissue (67). *EIF4E* gene copy number is increased in invasive carcinomas compared to normal tissues and benign tumors. The extent of *eIF4E* gene copy number increase is variable within each tissue category, and progression of malignant phenotype appears to be related to the extent of *eIF4E* gene copy number increase, which may be one of the mechanisms of eIF4E oncoprotein overexpression (68).

### 4.2 Increased stability of *eIF4E* mRNA

The changes in the stability of *eIF4E* mRNA may be related to the expression of eIF4E. *EIF4E* 3'UTR contains a unique AU-rich elements (AREs), which is responsible for binding *eIF4E* transcriptional stability regulators. HuR (69) and AUF1 p42 isoform are two factors that regulate transcriptional stability of *eIF4E* and competitively bind to the mRNA 3'UTR of *eIF4E*. In malignant tumor specimens with high expression of eIF4E, HuR is up-regulated, and its deletion leads to a decrease in *eIF4E* levels. With the increase of HuR level, the stability of *eIF4E* mRNA in cancer cells is improved. HuR and eIF4E regulate common transcripts involved in cell proliferation (Cyclin D1 and c-Myc) and neovascularization (VEGF). The p42 isoform of AUF1 interacts with the 3'UTR of *eIF4E* mRNA and decreases its stability (70).

### 4.3 Epigenetic modification

#### 4.3.1 Methylation

Protein arginine methyltransferase 5 (PRMT5) expression is significantly upregulated in a variety of cancer cells (71). Knockdown of PRMT5 decreases the methylation levels of H3 and H4 on promoter of *eIF4E*, thus reducing eIF4E expression (72–74). In colorectal cancer (CRC) tissues, the expression of eIF4E is positively correlated with PRMT5, silencing PRMT5 leads to decreased eIF4E expression (75). This helps to gain a deeper understanding of the overexpression of eIF4E in tumor cells.

#### 4.3.2 MiRNAs regulation

MiRNAs expression is frequently dysregulated in many diseases such as cancers and immune disorders, which is associated with the development of many pathological conditions and diseases (76). Down-regulation of miR-141 increases the expression of eIF4E, VEGF and c-Myc in H1299 or H2009 cells (77, 78). MiRNA-455-3p directly targets eIF4E and inhibits cap-dependent translation (79). On the other hand, miRNAs can recruit eIF4E2 to compete with eIF4E for binding to mRNAs, induce the dissociation of eIF4A from target mRNAs, block the assembly of the translation initiation complex, and thus inhibit mRNAs translation (80).

## 5 EIF4E and metabolic reprogramming

In 2011, Hanahan and Weinberg summarized immune evasion and metabolic reprogramming as the main characteristics of tumor cells (81). The overexpression of eIF4E plays an up-regulated role in both lipid metabolism and glycolysis, which suggests that overexpression of eIF4E can promote cancer by affecting metabolism.

### 5.1 EIF4E and glycolysis

Activation of mTORC1/eIF4E pathway promotes the translation of HIF-1α and up-regulation of HIF-1α can increase the expression levels of several glycolytic enzymes to drive glycolysis and produce more ATP (82, 83).

### 5.2 EIF4E and lipid metabolism

In 2012, about 3.9% (544300 cases) of cancers worldwide were related to obesity. In 2016, the International Agency for Research on Cancer concluded that there was a causal relationship between obesity and 13 cancers (84). Fang et al. reported that BMI is significantly positively correlated with endometrial cancer, esophageal adenocarcinoma and kidney cancer, while it is significantly negatively correlated with oral cavity cancer, lung cancer, premenopausal breast cancer and localized prostate cancer (85). Davide et al. showed that *eIF4E* single copy knockout mice gained only half the weight of normal mice under the same feeding conditions, suggesting that eIF4E is associated with obesity and that reducing eIF4E expression levels can enhance lipid metabolism and suppress obesity (86). Specifically, mRNAs involved in lipid storage pathways are translated in an eIF4E-dependent manner. Knockdown of *eIF4E*, which renders these mRNAs unable to be upregulated, leads to increased fatty acid oxidation and energy expenditure. Inhibition of eIF4E phosphorylation can suppress weight gain after intake of a high-fat diet. In conclusion, the translation control of eIF4E may be the driving factor of weight gain induced by high-fat diet and eIF4E can be used as a pharmacological target for obesity treatment (86).

## 6 Main factors affecting eIF4E activity

### 6.1 Intranuclear regulation of eIF4E

Both cytoplasmic and nuclear functions of eIF4E depend on its ability to bind to mRNA. Intranuclear regulation of eIF4E activity is mainly achieved by two proteins: PRH and PML, which are negative regulators of eIF4E-dependent mRNAs export (13, 87, 88).

PRH directly interacts with eIF4E through a conservative binding site, and inhibits the growth-promoting function of eIF4E by inhibiting its mRNAs transport function (61), and this is similar to that observed in the proline rich N-terminal region of eIF4G and 4EBP1 (62). PRH plays a major role in myelopoiesis, acting as both a tumor suppressor and an oncogene (60, 87). Alterations in the expression and intracellular localization of PRH are associated with breast, liver, thyroid cancers and certain leukemia subtypes. PRH is up-regulated in certain types of lymphoid leukemia (60), while it is down-regulated and eIF4E is up-regulated in some types of myeloid leukemia (AML, CML) (60, 87). In addition, PRH is a tissue-specific inhibitor of eIF4E-dependent Cyclin D1 mRNA transport (61). The level of Cyclin D1 is increased in leukemic specimens (87). Multiple PRH target genes including the genes encoding VEGF and VEGF receptors, are known to be important in the control of cell proliferation and cell survival (57). These findings suggest that the transport disorder of eIF4E mediated by PRH may be related to hematological malignancies.

PML is a potent inhibitor of eIF4E-dependent mRNAs export (62, 89), and is the first factor reported to modulate nuclear eIF4E functions (64). PML was shown to co-localize and co-immunoprecipitate with nuclear eIF4E (13). The RING domain of PML regulates the activity of eIF4E by directly interacting with the backside of eIF4E to drastically reduce (over 100 fold) the affinity of eIF4E for the 5' end cap structure of mRNAs (64, 88, 90–92). The back side of eIF4E is also the binding region for eIF4G and 4EBPs, suggesting that this region contributes to the positive and negative regulations of eIF4E activity (65). Overexpression of PML can inhibit AKT activity, leading to decreased phosphorylation levels of 4EBP1, which in turn affects cell survival, so PML may regulate eIF4E by inhibiting AKT activation (90).

Unlike the limited expression of PRH, PML is expressed in all mammalian cells and almost exclusively distributed in the nucleus, whereas eIF4E and PRH have a punctate distribution in the nucleus and the cytoplasm (61), but transport of eIF4E-dependent mRNAs is only inhibited by the nuclear part of PRH (87).

### 6.2 Phosphorylation of 4EBPs

The PI3K-AKT-mTOR signal transduction system significantly affects mRNAs translation by regulating the phosphorylation of its downstream targets. Growth factors, mitogens, and hormones can activate the PI3K-AKT-mTOR signaling pathway (93). Activated mTOR promotes the phosphorylation of 4EBPs (94). mTOR can form two distinct complexes, mTORC1 and mTORC2, which differ in their compositions, downstream targets, and sensitivity to rapamycin (95). The mTORC1 signaling pathway controls many cellular biological processes including mRNAs translation, cell growth and proliferation, and its downstream substrates mainly include 4EBPs and S6K (96). 4EBPs can regulate cell proliferation and S6K can regulate cell growth (97). mTORC1 sequentially phosphorylates 4EBP1 at T70 and S65, enabling the release of eIF4E to allow eIF4F assembly (54). Activated S6K can affect multiple factors involved in eIF4F assembly, promoting the recruitment of eIF4B to the pre-translational initiation complex for binding to eIF4A (98). mTORC2 controls cell survival by regulating the activity of AGC kinases, such as AKT, SGK1, and regulates the stability of nascent peptide chains. Studies have shown that more than 70% of cancer patients have excessive activation of mTOR (96).

A variety of factors can affect the phosphorylation of 4EBPs, such as endotoxin (LPS), insulin-like growth factor-1 protein (IGF-1) and branched-chain amino acids (99). LPS can transiently decrease phosphorylated 4EBP1 and increase the binding of 4EBP1 to eIF4E, leading to downregulation of IGF-1 expression (100), and 4EBP1 is inactivated by hyperphosphorylation in response to IGF-1 stimulation (101). In addition, O-glucoylation of 4EBP1 may regulate interaction between eIF4E and eIF4G, for example, when the level of O-glucoylation increases the binding of eIF4E to eIF4G decreases (102).

### 6.3 Phosphorylation and sumoylation of eIF4E

#### 6.3.1 Phosphorylation of eIF4E

The only kinases that phosphorylate eIF4E are mitogen-activated protein kinase interaction kinases MNK1 and MNK2 (103). The phosphorylation of eIF4E can stimulate the translation of tumor-promoting mRNAs, thus enhancing its carcinogenic characteristics (104).

RAS-RAF-MEK-ERK-MNK and ASK1-MKK3/6-p38-MNK cascade reactions activate transcription factors and regulate gene expression. The cascade involves three upstream kinases, namely mitogen-activated protein kinase (MAPK), MAPK kinase (MAPKKs/MEK/MKK) and MAPKK kinase (MAPKKKs). The three kinases integrate upstream signals and transmit them to a series of downstream effectors. MAPK pathway has four main branches, namely extracellular signal-regulated protein kinase (ERK), p38 mitogen-activated protein kinase (p38 MAPK), c-Jun N-terminal kinase (JNK) and extracellular signal-regulated protein kinase 5 (ERK5) (105). MEK1 and MEK2 are activators of ERK1/2, MEK5 is an activator of ERK5, MKK4 and MKK7 are activators of JNK, and MKK3 and MKK6 are activators of p38 MAPK.

##### 6.3.1.1 RAS-RAF-MEK-ERK-MNK pathway

**Figure 6:**
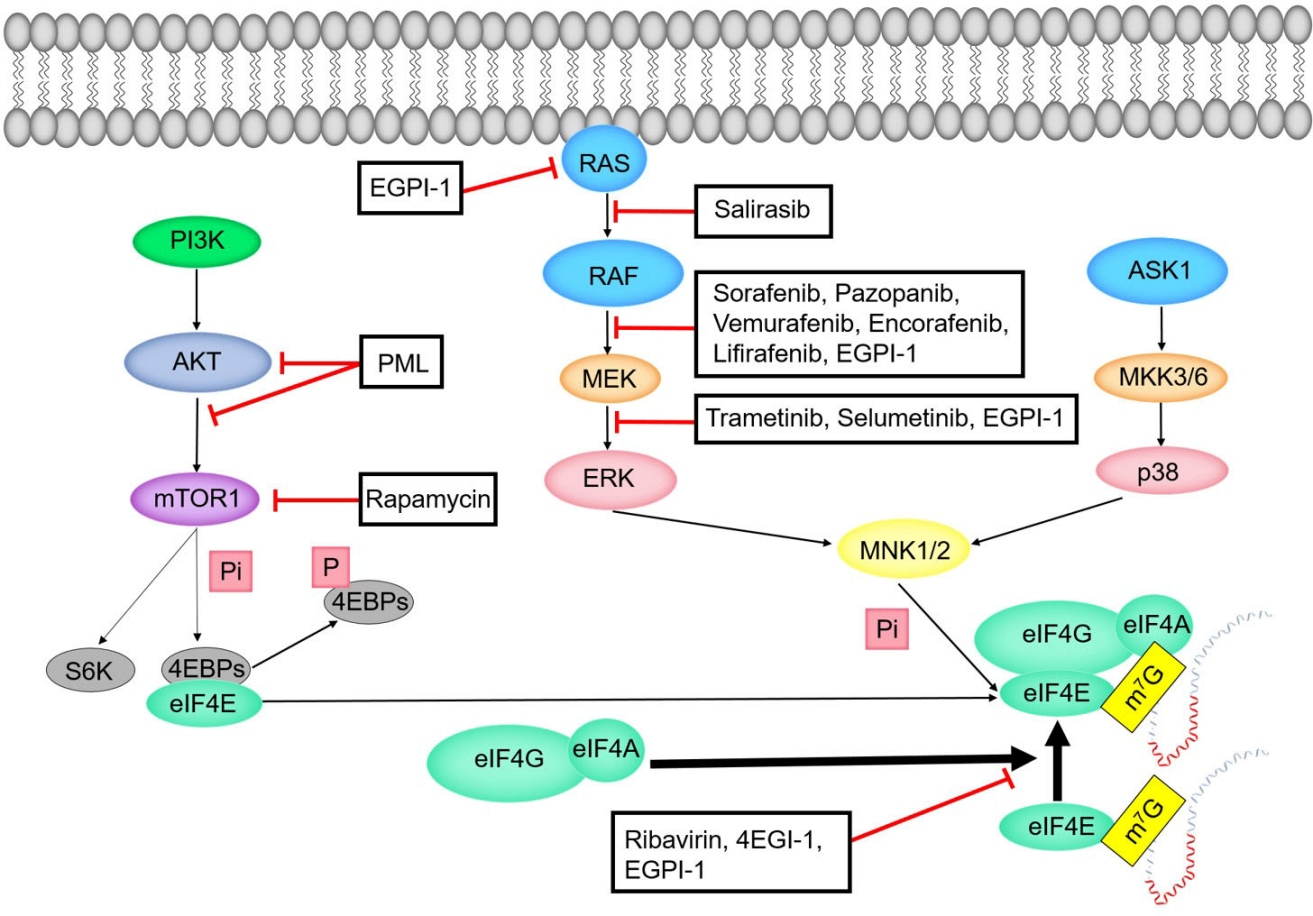
Regulation mechanism of eIF4E activity.

The process of phosphorylation of eIF4E by RAS-RAF-MEK-ERK-MNK pathway can be divided into five stages (**Figure 6**). In the first stage, RAS protein recruits and activates protein kinase RAF. RAS protein is related to many physiological processes such as cell metabolism, cell proliferation and apoptosis (106). RAS is often activated in a variety of human cancers (106). About 19% of cancer patients have RAS mutations (107). The RAS mutation that causes the constitutive activation of MAPK pathway is the most common mutation in human cancers (108). In the second stage, RAF, as MAPKKK, starts the activity of MEK by phosphorylating the activated fragment of MEK. In the third stage, MEK transmits signals by phosphorylating the activated fragment of ERK1/2. ERK1 and ERK2 belong to the mitogen activated protein kinase family. MAPK/ERK pathway is related to cell proliferation, differentiation, migration, aging and apoptosis (105). In the fourth stage, activated ERK1/2 is transferred to the nucleus to phosphorylate a variety of substrates including transcription factors (108–110). In the fifth stage, MNK1 and MNK2 phosphorylate the conserved site S209 of eIF4E after they were activated by ERK and p38 (103, 111). When eIF4E is combined with the N-terminal of eIF4G, MNK1/MNK2 is combined with the C-terminal of eIF4G. In other words, MNK phosphorylates S209 of eIF4E with eIF4G as the docking site (112, 113).

##### 6.3.1.2 ASK1-MKK3/6-p38-MNK pathway

The process of phosphorylation of eIF4E by ASK1-MKK3/6-p38-MNK pathway can be divided into four stages (**Figure 6**). In the first stage, apoptosis signal regulated kinase 1 (ASK1) is the MAPKKK of JNK and p38 MAPK pathways. ASK1 is preferentially activated by various cytotoxic stressors and plays a key role in various cellular responses (114). In the second stage, ASK1 activates two different subgroups of MAPKK, SEK1 (or MKK4) and MKK3/MKK6. In the third stage, MKK3/MKK6 activates the p38 subgroup of MAPK (115, 116). In the fourth stage, p38 MAPK activates MNK to phosphorylate eIF4E.

#### 6.3.2 Sumoylation of eIF4E

Many targets related to sumoylation are linked to transcription, DNA repair and nucleocytoplasmic transport. Sumoylation of eIF4E can enhance its translation activity (117) and promote the formation of active eIF4F, and its destruction can inhibit the translation of eIF4E-dependent mRNAs. More than 20% of eIF4E combined with cap structure of mRNAs are sumoylated, which indicates that sumoylation may be crucial to the functions of eIF4E at the initiation of translation. Sumoylation does not directly affect the cap binding activity of eIF4E, but promotes the dissociation of eIF4E with 4EBP1. At the molecular level, sumoylation may regulate the function of proteins by changing their conformations. The conformational change of eIF4E may increase its affinity with proteins that have a certain conformation (such as eIF4G), and decrease its affinity with 4EBP1 and other proteins. Phosphorylation of S209 is necessary for eIF4E sumoylation (117, 118), while phosphorylation of eIF4E does not require its sumoylation. Histone deacetylases (HDACs) induce the formation of active eIF4F complexes by stimulating eIF4E sumoylation (119).

## 7 EIF4E and tumorigenesis 

EIF4E plays a crucial role in the malignant transformation, progression and drug resistance of many human solid tumors (120). When tissue cells are stimulated by growth factors, mitogens, et al., multiple pathways work together to increase the expression and activity of eIF4E. Overexpression of eIF4E can selectively enhance the translation of weak mRNA that is not expressed or weakly expressed in normal cells, resulting in the abnormal expression of these genes related to cell cycle, vascular growth and cell survival. Then the translation of multiple essential proteins in the process of tumor occurrence and development is increased, promoting cell proliferation and malignant transformation, increasing the malignant degree of tumor tissues, facilitating the formation of tumor blood vessels and thus providing more nutrition for tumor tissues, which is conducive to the growth, invasion and metastasis of tumor cells. There is a common mechanism for eIF4E in different cancers: eIF4E mediates normal cell proliferation, but induces tumorigenesis when it is dysregulated and over expressed (121). The expression of eIF4E in tumor tissue is significantly higher than that in normal tissue. With the progress of cancer, the expression of eIF4E is increasing. In addition, overexpression of eIF4E will increase the probability of cancer recurrence. Therefore, eIF4E is a marker of tumor progression, malignant transformation, metastasis, and poor prognosis. It is also a sensitive molecular marker in tumor detection (**Table 2**).

**Table 2:** The hazard ratio of eIF4E in various cancers.

Cancer	Disease progression	Positive rate
Laryngeal carcinoma (122)	10mm incision margin of supraglottic carcinoma	22.20%
	5mm incision margin of supraglottic carcinoma	50.90%
	Supraglottic carcinoma	91.30%
	10mm incision margin of hypopharyngeal carcinoma	58.82%
	5mm incision margin of hypopharyngeal carcinoma	82.35%
	Hypopharyngeal carcinoma	86.70%
Squamous cell carcinoma of lung (123)	Normal lung tissue	30.00%
	Paracancerous tissue	50.00%
	Squamous cell carcinoma of lung	75.00%
Breast cancer (124)	Paracancerous tissue	10.00%
	Breast cancer	87.50%
Cervical carcinoma (125)	Chronic cervicitis	9.50%
	Cervical intraepithelial neoplasia I	40.00%
	Cervical intraepithelial neoplasia II-III	61.30%
	Invasive carcinoma	90.50%
Nasopharyngeal carcinoma (126)	Chronic inflammatory tissue of nasopharyngeal mucosa	10.00%
	Advanced nasopharyngeal carcinoma tissue	66.67%
Gastric cancer (127)	Chronic non-atrophic gastritis	0
	Chronic atrophic gastritis	16.70%
	Low grade intraepithelial neoplasia	20.00%
	High grade intraepithelial neoplasia	59.30%
	Gastric cancer	91.80%
Renal clear cell carcinoma (128)	Paracancerous tissue	27.50%
	Renal clear cell carcinoma	77.50%

Positive means eIF4E is over expressed, negative means eIF4E is not over expressed.

### 7.1 Expression of eIF4E in laryngeal carcinoma

Franklin et al. showed that eIF4E in the tumor area of patients was over expressed, and the expression of FGF2 and VEGF was increased. The overexpression of eIF4E at the incisal edge would increase the probability of tumor recurrence (129). Liang et al. found that eIF4E was over expressed in laryngeal squamous cell carcinoma samples, but not in vocal cord polyps samples. Overexpression of eIF4E led to up regulation of bFGF. EIF4E and bFGF played a synergistic role in the genesis, development, invasion and metastasis of laryngeal squamous cell carcinoma (130). Yi et al. detected tumor markers such as Cyclin D1, p53 and eIF4E at the surgical margin, and the results showed that eIF4E was the most sensitive molecular indicator among these molecules (131).

### 7.2 Expression of eIF4E in lung cancer

Chan et al. found that the overexpression of 4EBP1 led to the decrease of eIF4E, and the expression of FGF2 and VEGF was inhibited, which could inhibit the proliferation of K-RAS^LA1^ mouse lung cancer cells (132). According to Dong et al., the expression of eIF4E in the serum of NSCLC patients was significantly higher than that in healthy individuals, and eIF4E was an independent prognostic factor for shortening overall survival and progression free survival (133). According to Qi et al., eIF4E was highly expressed in multiple lung cancer cell lines, and siRNA-eIF4E could significantly inhibit lung cancer cell proliferation (134). Yoshizawa et al. showed that the overexpression rate of p-eIF4E in 300 NSCLC tissues was 39.9%, suggesting that the activity of eIF4E in NSCLC was increased (135).

### 7.3 Expression of eIF4E in breast cancer

Kerekatte et al. showed that eIF4E was overexpressed in breast carcinomas, but not in normal breast tissue and benign breast lesions (hyperplasia or inflammation) (136). Derek et al. believed that the expression level of eIF4E was related to tumor recurrence, and eIF4E might increase the risk of recurrence of breast cancer (137). Avdulov et al. found that the level of p-4EBP1 in breast cancer cells was increased, and the assembly ability of eIF4F was enhanced (53). Yang et al. showed that the overexpression of eIF4E was related to the formation of various human malignant tumors, including breast cancer (138). RNA interference system driven by the Survivin promoter efficiently and specifically downregulated eIF4E expression in human breast cancer cells but not in normal human breast epithelial cells. Therefore, RNAi driven by Survivin promoter targeting eIF4E could be used as an adjuvant therapy tool for human breast cancer, with tumor specificity and efficiency (120).

### 7.4 Expression of eIF4E in head and neck squamous cell carcinoma

Haydon et al. found that the amplification and overexpression of *eIF4E* gene gradually increased from normal head and neck tissues to benign tumors, and ultimately to invasive cancer cells in the head and neck. This expression characteristic of eIF4E makes it a potential tumor detection indicator (68). Patients with head and neck squamous cell carcinoma (HNSCC) have a high risk of metastasis and recurrence, and aberrant activation of PI3K/AKT/mTOR signaling occurs in approximately 80% of HNSCC, which has been suggested as a prognostic biomarker for patients with recurrence or metastasis (139). The expression of eIF4E was elevated in HNSCC, and its overexpression made VEGF and FGF2 preferentially up-regulated. Inhibition of eIF4E expression by antisense RNA could reduce the tumorigenicity and angiogenesis of cells. Antisense RNA therapy of eIF4E might be used as an adjuvant treatment for head and neck cancer, especially when eIF4E was found to be elevated at the surgical margin (140). The expression of eIF4E and Cyclin D1 increases in HNSCC, both of which could stimulate cell cycle progression and transform squamous epithelial cells (141). In HNSCC, eIF4E seems to be a more significant prognostic indicator than p53 (142). The expression of eIF4E relative to 4EBP1 is a more precise predictor of HNSCC and its progression (143).

### 7.5 EIF4E and colorectal carcinoma

Niu et al. showed that eIF4E was an indicator of tumor progression and poor prognosis in colon cancer patients (144). Gao et al. found that eIF4E was up-regulated in CRC, and its expression frequency (EF) in cancer tissues was higher than that in normal adjacent tissues (145). Ruan et al. showed that eIF4E could significantly affect CRC organoid growth (146).

### 7.6 EIF4E and leukemia

The elevation of eIF4E level will lead to the imbalance of eIF4E-dependent mRNAs transport, which will hinder the differentiation of granulocytes and monocytes, especially in myeloid leukemia and may contribute to the occurrence of leukemia. This abnormality of eIF4E is caused by the imbalance of PRH (87).

In acute myeloid leukemia (AML), the increased expression and phosphorylation of eIF4E are associated with poor prognosis, and phosphorylated eIF4E can be used as a prognostic indicator and potential anti-cancer target for biological therapy of AML (147, 148). In chronic myeloid leukemia (CML), the expression and phosphorylation level of eIF4E are up-regulated, and the activity of eIF4E in patients with advanced CML is significantly increased. In addition, abnormal activation of eIF4E in leukemic stem cells of CML in blast phase significantly increases the synthesis of β-Catenin (149).

Changes of many signal pathways have been found in acute lymphoblastic leukemia (ALL), including RAS-RAF-MEK-ERK and PI3K-AKT-mTOR pathways (150). Inhibition of eIF4E may be a new method to treat pre-B cell leukemia while preserving the development and function of normal B cells (7).

## 8 Targeted therapy of eIF4E

**Figure 7:**
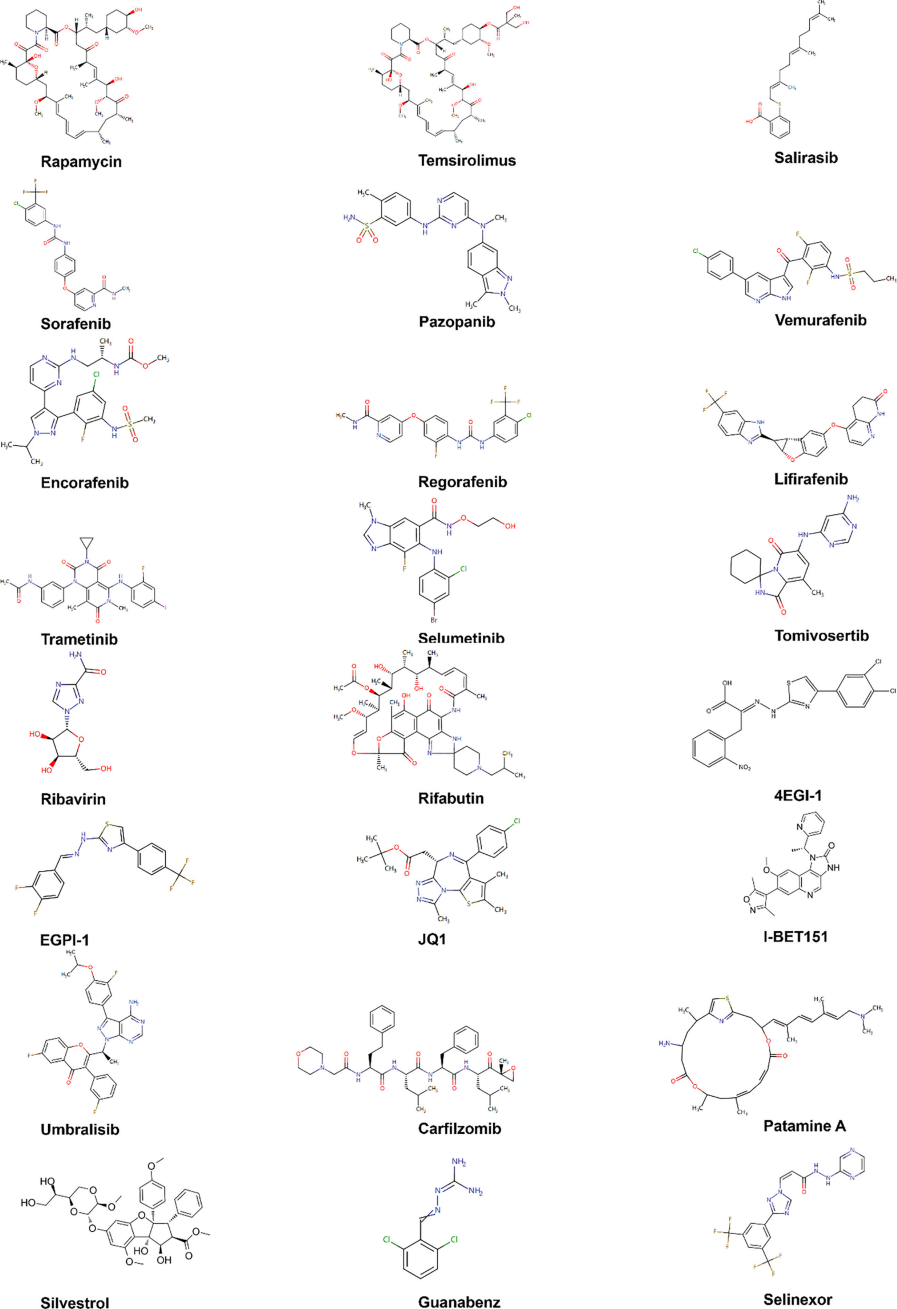
Chemical structures of inhibitors. Chemical structures are sourced from the DrugBank database (https://go.drugbank.com/).

EIF4E specific antisense oligonucleotide (4EASO) is a new drug that can reduce the level of eIF4E and the formation of eIF4F complex (151, 152). 4EASO can effectively down-regulate the expression of eIF4E protein in breast and prostate tumor xenografts (153), significantly inhibits tumor growth by binding to eIF4E mRNA, triggering RNA degradation mediated by RNA enzyme H (153). The early generation of ASO lacks nuclease resistance and tissue stability, resulting in a relatively short half-life and significantly reduced efficacy. In contrast, the efficacy, nuclease resistance and tissue half-life of the second generation ASO were significantly improved (154). LY2275796 is a second-generation antisense anticancer drug (**Figure **7 and **Table 3**).

**Table 3:** Inhibitors of eIF4E activation pathway.

Drugs	Targets	Mechanisms	Related diseases	Clinical trials
LY2275796	eIF4E	Complementing with eIF4E mRNA, it promotes RNA degradation mediated by RNA enzyme H and blocks the translation of eIF4E mRNA.	Cancer/tumor (unspecified) and solid tumor	Phase I
Rapamycin	mTOR	It plays an immunosuppressive role by inhibiting the activation and proliferation of T cells. It combines with FKBP12 to form rapamycin-FKBP12 complex, which can inhibit the activity of mTOR.	Hysteromyoma	Phase IV
			Squamous cell skin cancer	Phase II
			Solid tumor	Phase I
Temsirolimus (155)	mTORC1	It specifically binds to the C-terminal FRB region of mTOR, prevents mTOR from modifying downstream target proteins, and inhibits the proliferation and growth of tumor cells by participating in the regulation of various substances metabolism.	Non Hodgkin’s lymphoma	Phase IV
			Hepatoblastoma, malignant lymphoma, mantle cell lymphoma, tumor, renal cell carcinoma, bladder cancer	Phase III
			Breast cancer, myeloma, non-small cell lung cancer	Phase II
Salirasib	RAS	It is a FTS simulator, which simulates the carboxyl terminal FTS related structure shared by all RAS proteins, and removes the active RAS proteins from the cell membrane (156, 157)	Pancreatic cancer, non-small cell lung cancer (158)	Phase II
Sorafenib	RAF	It has the activity of inhibiting RAF kinase and several receptor tyrosine kinases, inhibits VEGF, and downregulates the anti-apoptotic protein Mcl-1 in a MEK/ERK independent manner (159–161)	Hepatocellular carcinoma, renal cell carcinoma (159–161)	Phase IV
			Acute myeloid leukemia, granulocytic sarcoma, skin leukemia, bone marrow tumor, fibromatosis desmoidosis, breast cancer, non-small cell lung cancer, melanoma	Phase III
			Differentiated thyroid carcinoma, gastrointestinal stromal tumor	Phase II
Pazopanib		Targeting VEGFR1, VEGFR2, VEGFR3, PDGFRα, PDGFRβ And c-kit as a vascular endothelial growth factor receptor inhibitor, it can act directly on tumor cells as a RAF inhibitor (162, 163)	Metastatic clear cell renal cell carcinoma, adult soft tissue sarcoma	Phase IV
			Renal cell carcinoma, ovarian carcinoma, sarcoma	Phase III
Vemurafenib		As a competitive inhibitor of BRAF mutants, it is particularly effective for BRAF V600E mutation (164, 165). Vemurafenib blocks the downstream process to inhibit tumor growth and ultimately trigger apoptosis.	Malignant melanoma (164, 165)	Phase IV
			Biliary duct cancer/bladder cancer, cancer/tumor/salivary cancer/solid tumor	Phase II
Encorafenib		It plays a role in regulating MAP kinase/ERK signaling pathway, thus affecting cell division, differentiation and secretion.	Solid tumor	Phase IV
			Melanoma (166, 167), biliary tract tumor, metastatic colorectal cancer	Phase III
Regorafenib		It is a small molecule inhibitor of various membrane binding and intracellular kinases and participates in normal cell functions and pathological processes.	Metastatic colorectal cancer and gastrointestinal stromal tumor (168)	Phase IV
			Gastroesophageal cancer, liver cancer	Phase III
Lifirafenib		Lifirafenib effectively inhibits RAF kinase family and EGFR activity.	Adult solid tumor (169, 170)	Phase II
Trametinib	MEK1/2	It is a reversible allosteric inhibitor, which can reverse the activation of MEK1 and MEK2 and the activity of MEK1 and MEK2 kinase.	Melanoma (171), high grade glioma, non-small cell lung cancer, rare cancer, solid tumor	Phase IV
			Soft tissue sarcoma, differentiated thyroid carcinoma	Phase III
Selumetinib		Regulate the level of MEK.	Metastatic/uveal melanoma, low-grade glioma, neurofibromatosis type 1, glioma of visual pathway, differentiated thyroid cancer	Phase III
			Adenocarcinoma, non-small cell lung cancer, squamous cell carcinoma, cholangiocarcinoma, gallbladder carcinoma	Phase II
Tomivosertib (eFT508)		By selectively inhibiting MNK1/2, it plays a role in multiple nodes of the cancer immune cycle, selectively inhibiting eIF4E phosphorylation, thus promoting fat burning (86).	Lymphoma, solid tumor, non-small cell lung cancer, hepatocellular carcinoma, triple negative breast cancer, advanced solid tumor and recurrent or refractory microsatellite stable colorectal cancer	Phase II
Ribavirin (155)	eIF4E	It directly binds to eIF4E and competitively inhibits the binding of eIF4E to the 5' end cap of mRNAs, thereby inhibiting mRNAs output and translation function of eIF4E.	Hematologic malignancies, liver cancer	Phase IV
			Acute myeloid leukemia (172), breast cancer, oropharyngeal cancer, prostate cancer and other solid tumors	Phase II
Rifabutin	eIF4E, β protein	Rifabutin plays a role in lung cancer cells by targeting eIF4E and β-Catenin.	Lung cancer	——
4EGI-1	eIF4E	It has high affinity for eIF4E and can inhibit the interaction between eIF4E and eIF4G as well as protein and protein (173, 174), leading to the blocking of eIF4E binding to eIF4G.	Leukemia (173), lung cancer, multiple myeloma, breast cancer (175)	——
EGPI-1	RAS, eIF4E, 4EBP1	The expression of RAS, p-MNK, p-ERK and p-eIF4E were significantly inhibited by EGPI-1. It interferes with the interaction between eIF4E and eIF4G, inhibits 4EBP1 phosphorylation, destroys mitochondrial function through mTOR/4EBP1 signal pathway, and induces autophagy, apoptosis and reactive oxygen species generation (134).	Lung cancer	——
JQ1	eIF4E	JQ1 and I-BET151 reduce eIF4E transcription and subsequent mRNAs and proteins expression by inhibiting BET (176, 177).	Lung cancer (176, 177)	——
I-BET151				
Umbralisib (155)	4EBP1	Umbralisib and carfilzomib synergistically inhibit the phosphorylation of 4EBP1 and the translation of *c-myc*.	Recurrent diffuse large B-cell lymphoma, follicular lymphoma, mantle cell lymphoma	Phase II
			Recurrent and refractory marginal cell lymphoma and follicular lymphoma in adults	Phase I
Carfilzomib (155)			Recurrent or refractory multiple myeloma	Phase IV
			Multiple myeloma, plasma cell myeloma	Phase III
			Mantle cell lymphoma	Phase II
Patamine A (155, 178)	eIF4A	Patamine A and silvestrol promote the RNA binding ability, ATPase and helicase activity of eIF4A, leading to the removal of eIF4A from eIF4F complex through RNA mediated eIF4A isolation.	Melanoma, non-small cell lung cancer and colon cancer	——
Silvestrol (155, 178)		Silvestrol induces autophagy and Caspase mediated apoptosis.	Melanoma, breast cancer and prostate cancer, chronic/acute lymphoblastic leukemia, acute myeloid leukemia, hepatocellular carcinoma, brain cancer (meningioma)	——
Guanabenz (178)	eIF2α	It is a type of selective agonist for α-2 adrenoceptor, often used as antihypertensive drug, inhibiting dephosphorylation of eIF2α induced by stress.	Hypertension and parasitic disease	——
Selinexor (155)	XPO1	It blocks the nuclear output of tumor suppressors (p53, p21 and BRCA1/2) mediated by XPO1.	Multiple myeloma, endometrial carcinoma	Phase III
			Acute myeloid leukemia, breast cancer	Phase II

Rapamycin is an FDA approved antibiotic and immunosuppressant against mTOR, which can inhibit the kinase activity of mTOR1. Unlike rapamycin analogues, ATP competitive mTOR inhibitors target ATP binding sites (179), weakening the activity of mTORC1 and mTORC2 (179, 180). At present, many clinical trials of ATP competitive mTOR inhibitors for malignant tumors are under way (179).

Trametinib is a clinically approved anti MEK drug, but its efficiency is low, which may be due to the rapid development of drug-resistant diseases. Trametinib’s transience is attributed to negative feedback loop inhibition, which reactivates MAPK pathway through various compensatory mechanisms (181). For some xenografts, the combined treatment of trametinib and pazopanib resulted in a continuous reduction of tumor volume by 50% or more (182).

The anti-cancer drug eFT508 (tomivosertib) developed by Davide et al. selectively blocked the phosphorylation of eIF4E by targeting inhibition of MNK1/2, thus inhibiting the weight gain after eating a high-fat diet. Studies showed that eFT508 alone can enhance anti-tumor immunity. This also supports the statement that there is a connection between eIF4E, obesity and cancer development (86, 183).

Ribavirin competitively inhibits the binding of eIF4E to the 5' end cap of mRNAs, as well as has an anti-proliferation effect. It can down regulate the phosphorylation levels of AKT, mTOR, 4EBP1 and eIF4E proteins in the mTOR-eIF4E signal pathway, as well as the phosphorylation levels of MEK, ERK, MNK1 and eIF4E proteins in the ERK-MNK1-eIF4E signal pathway. Ribavirin significantly increased the binding of eIF4E to 4EBP1, and decreased the binding of eIF4E to eIF4G. Ribavirin effectively targets eIF4E of leukemia patients with poor prognosis, leading to significant clinical reactions including complete remission and partial remission (148). The combination of ribavirin and imatinib can enhance the anti-leukemia effect (184).

EIF4E and β-Catenin are key regulators of the growth and survival of lung cancer cells, and their pharmacological inhibition may have therapeutic effects on lung cancer (185). Phosphorylation of eIF4E S209 can activate β-Catenin (186). Rifabutin inhibits eIF4E phosphorylation, leading to the decreased phosphorylation of β-Catenin and its subsequent transcriptional activity. The absence of eIF4E eliminates the effect of rifabutin on the inhibition of β-Catenin activity, which further confirms that rifabutin plays a role in lung cancer cells by targeting eIF4E and β-Catenin. The overexpression of β-Catenin reverses the inhibition of rifabutin on cell growth and survival.

EIF4E regulates gene translation and has been proved to play an important role in the progression of lung cancer. BET protein can regulate gene transcription. BET inhibitors JQ1 and I-BET151 inhibit the growth of NSCLC and down regulate the expression of eIF4E. When *BRD4*, a member of the BET family, was knocked out by siRNA, the growth of NSCLC was inhibited and the level of eIF4E protein was reduced. In addition, overexpression of eIF4E partially eliminated the growth inhibition function of JQ1, while knockout of *eIF4E* enhanced the growth inhibition function of JQ1. This indicates that JQ1 and I-BET151 inhibit the growth of NSCLC by inhibiting BET to reduce the transcription and subsequent protein expression of eIF4E (176, 177).

4EGI-1, a small molecular compound targeting to inhibit eIF4E-eIF4G binding, can reduce the expression of c-Myc without affecting the expression of β-actin (173) and has been proved to play an anti-cancer role in human cancer cells (187) without obvious toxicity (175).

EGPI-1 is a small molecule compound that can induce the down-regulation of eIF4E downstream proteins such as c-Myc. Further studies showed that EGPI-1 could significantly inhibit the proliferation of various lung cancer cells, such as A549, NCIL-H460, NCI-H1650 and 95D, but did not affect the proliferation of HUVEC cells (134). EGPI-1 showed good safety and pharmacokinetics characteristics *in vivo*. These results indicate that EGPI-1 can be used as an excellent lead compound to develop new anti-cancer drugs targeting eIF4E-eIF4G interface, and also as a chemical genetic probe to study the mechanism of eIF4E in biological processes and human diseases (188).

## 9 Outlook

This review mainly introduces eIF4E from its structure, biological function, main factors affecting its activity, and targeted drugs. However, there is still a lot of content not covered in this paper.

EIF4E mediates the proliferation of normal cells, but induces tumorigenesis when over expressed. Maintaining the level of eIF4E below its cancer promoting threshold is an important anti-cancer measure for normal cells (189). First of all, for future clinical trials, we should focus on setting up prospective randomized controlled studies to analyze the levels of patients' eIF4E and 4EBPs as indicators of disease detection, so as to formulate effective treatments at the early stage of the disease. In addition, the exact mechanism of tumor occurrence, development, and metastasis mediated by eIF4E is still poorly understood, and the relationship between eIF4E and tumors should be paid attention to and further studied. The sensitivities of different tumors to the same drug are also different, and the reasons for this difference deserve further in-depth study in order to achieve efficient treatment and reduce side effects. Finally, using eIF4E as a molecular target for tumor therapy, establishing models that multiple anti-tumor drugs are used in combination may provide new ideas for improving the efficacy and reducing side effects. For example, trametinib and pazopanib, umbilisib and carfilzomab, as well as patamine A and silvestrol can be used in combination to achieve anti-tumor effects.

To sum up, eIF4E will become an important target for the treatment of tumors in the future, and as a highly sensitive biomarker in tumor detection, it will be used for early diagnosis and evaluation of prognosis, opening up broad prospects for the treatment of malignant tumors.

## Author contributions

Conception: LL and BX. Writing-original draft: XC, LL, and BX. Methodology, investigation and revising: YA, MT, and DX. Writing-Reviewing and Editing: XC, LL, and BX. All authors listed have made substantial, direct and intellectual contributions to this work, and approved it for publication.
